# Investigating the Contribution of Peri-domestic Transmission to Risk of Zoonotic Malaria Infection in Humans

**DOI:** 10.1371/journal.pntd.0005064

**Published:** 2016-10-14

**Authors:** Benny O. Manin, Heather M. Ferguson, Indra Vythilingam, Kim Fornace, Timothy William, Steve J. Torr, Chris Drakeley, Tock H. Chua

**Affiliations:** 1 Department of Pathobiology and Medical Diagnostics, Faculty of Medicine and Health Sciences, Universiti Malaysia Sabah, Kota Kinabalu, Sabah, Malaysia; 2 Institute of Biodiversity, Animal Health and Comparative Medicine, University of Glasgow, United Kingdom; 3 Parasitology Department, Faculty of Medicine, University of Malaya, Kuala Lumpur, Malaysia; 4 Faculty of Infectious and Tropical Diseases, London School of Hygiene and Tropical Medicine, London, United Kingdom; 5 Jesselton Medical Centre, Kota Kinabalu, Sabah, Malaysia; 6 Department of Vector Biology, Liverpool School of Tropical Medicine, United Kingdom; University of Florida, UNITED STATES

## Abstract

**Background:**

In recent years, the primate malaria *Plasmodium knowlesi* has emerged in human populations throughout South East Asia, with the largest hotspot being in Sabah, Malaysian Borneo. Control efforts are hindered by limited knowledge of where and when people get exposed to mosquito vectors. It is assumed that exposure occurs primarily when people are working in forest areas, but the role of other potential exposure routes (including domestic or peri-domestic transmission) has not been thoroughly investigated.

**Methodology/Principal Findings:**

We integrated entomological surveillance within a comprehensive case-control study occurring within a large hotspot of transmission in Sabah, Malaysia. Mosquitoes were collected at 28 pairs households composed of one where an occupant had a confirmed *P*. *knowlesi* infection within the preceding 3 weeks (“case”) and an associated “control” where no infection was reported. Human landing catches were conducted to measure the number and diversity of mosquitoes host seeking inside houses and in the surrounding peri-domestic (outdoors but around the household) areas. The predominant malaria vector species was *Anopheles balabacensis*, most of which were caught outdoors in the early evening (6pm - 9pm). It was significantly more abundant in the peri-domestic area than inside houses (5.5-fold), and also higher at case than control households (0.28±0.194 vs 0.17±0.127, p<0.001). Ten out of 641 *An*. *balabacensis* tested were positive for simian malaria parasites, but none for *P*. *knowlesi*.

**Conclusions/Significance:**

This study shows there is a possibility that humans can be exposed to *P*. *knowlesi* infection around their homes. The vector is highly exophagic and few were caught indoors indicating interventions using bednets inside households may have relatively little impact.

## Author Summary

The primate knowlesi malaria has now emerged in human populations throughout South East Asia. Our limited knowledge of where and when people get exposed to the vector (*Anopheles balabacensis*) has resulted in poor control measures, although it is assumed that exposure occurs primarily when people are working in forest areas. We investigated the role of peri-domestic (outdoors but around the household) and domestic transmission. Mosquitoes were collected at 28 pairs households composed of one where an occupant had a confirmed knowlesi malaria infection and an associated “control” where no infection was reported. Most of the vectors were caught outdoors from 6pm - 9pm. The vectors were also significantly more abundant in the peri-domestic area than inside houses (5.5-fold), and also higher at case than control households. Ten *Anopheles* (out of 641) were found positive for primate malaria parasites. This study shows that humans can be exposed to knowlesi infection around their homes. Given the vectors are mainly outdoor biters, interventions using insecticide treated bednets inside households may have relatively little impact. A paradigm shift in control methods is required to reduce infection of this primate malaria.

## Introduction

The success story of reducing malaria worldwide [1] has been marred by a few notable exceptions where bulk of disease is caused by zoonotic “neglected” malaria species with atypical transmission that makes them less easy to control. Zoonotic malaria, such as *Plasmodium knowlesi* from the long tailed macaque (*Macaca fascicularis*) in SE Asia [2], and *P*. *brasilanum* from NewWorld monkeys in South America [3], are a growing public health problem.

In South East Asia, the long tailed macaque harbours at least five simian malarias, namely, *P*. *coatneyi*, *P*. *inui*, *P*. *fieldi*, *P*. *cynomolgi* and *P*. *knowlesi* [4,5]. *Plasmodium knowlesi* is presently the main zoonotic malaria with the greatest public health importance, especially in Sabah, Malaysian Borneo which has recorded the highest growing number of *P*. *knowlesi* cases in humans, and most of these cases are clustered within one district, Kudat in the North eastern region [6,7]. *Plasmodium knowlesi* is morphologically similar to *P*. *malariae* and had been misdiagnosed as such for a long time [2]. A first case of naturally acquired human infection with *P*. *cynomolgi* has also been reported from peninsular Malaysia [8]. Thus it is a possibility that other primate parasites may also be soon contributing to human cases as previously predicted [9].

In Sabah, it has been confirmed that *An*. *balabacensis* is the primary vector [10] and the long tail macaques, which are the natural reservoir hosts for simian malaria parasites are present. Furthermore, Kudat district has many secondary forest areas surrounded by hilly areas, oil palm estates and rubber plantations which in general serve as habitats not only for long-tail macaques but also for *Anopheles* species. Close interaction between monkeys, mosquitoes and human increases the chances of being infected with *P*. *knowlesi*.

The vectors of *P*. *knowlesi* malaria in Malaysia comprise of five *Anopheles* species of the Leucospyrus group namely: *An*. *hackeri*, *An*. *latens*, *An*. *cracens*, *An*. *introlatus* and *An*. *balabacensis* [10–15]. In Vietnam *Anopheles dirus* of the Dirus group was recorded as the vector [16,17]. These vectors are found mainly in the forests and are outdoor biters, and likely to have low susceptibility to frontline control strategies which typically involve use of insecticides in homes.

In Malaysia, the National Malaria Eradication Program was launched in 1967, followed by state-wide malaria control programs during the 1970s and 1980s. Consequently, great reductions in malaria prevalence were recorded, from 240,000 in 1961 to around 50,000/year during the 1980s [11,18]. The success of the eradication programs was also reflected in Sabah, East Malaysia where malaria notifications decreased sharply, from peak notifications of 33,153 and 15,877 during 1994–1995 for *P*. *falciparum* and *P*. *vivax* respectively to 605 and 628 respectively in 2011. Similarly notifications of *P*. *malariae/P*. *knowlesi* also fell from a peak of 614 in 1994 to <100/year in the late 1990s/early 2000s [6].

Although Malaysia has shown considerable success in the control of human malaria and is on target towards elimination of malaria by 2020 [19], notifications of suspected *P*. *knowlesi* cases have increased from 59 notifications in 2004 to 996 in 2013, an overall increase of over 16-fold [6]. According to the Malaysian Ministry of Health, *P*. *knowlesi* is the predominant species occurring in the country comprising 62% of the cases in 2013 [20]. It was suggested that the increase in number of *P*. *knowlesi* notification in Kudat maybe be due to high awareness of knowlesi infection among physicians and availability of better diagnostic tools to identify this malaria parasite [20]. In other words what is reported now represents the true infection rate as compared in the late 1990s and early 2000s when *P*. *knowlesi* cases were misdiagnosed as *P*. *malariae*. However, recent findings by Fornace et al. had demonstrated a clear link between land use change and *P*. *knowlesi* incidence, which strongly supports the idea that this is not just a problem of poor diagnosis/changing awareness, but a real epidemiological change [21].

The frontline vector control methods practiced in Malaysia under the malaria elimination programme are same as those used in several other endemic settings within the region i.e. the application of insecticides in houses either through use of Long Lasting Insecticide Treated Nets (LLINs) or Indoor Residual Spraying (IRS). However there is little evidence to support that this is effective against *P*. *knowlesi* vectors.

Thus there is a need for more detailed entomological investigation to assess the relative importance of exposure to mosquito vectors at or away from home, and to design control measures accordingly. Our working hypothesis is that given *An balabacensis* is the primary vector, we would expect that infection risk is higher when they are present. Towards this end, a case-control entomological study was conducted to determine if *P*. *knowlesi* infection risk is linked to exposure to vectors in domestic and peri-domestic settings. Specific comparisons were included to test for differences in vector abundance, species composition, biting time and infection rate at case and control households. Further aims were to assess and characterize the biting behavior of *P*. *knowlesi* vectors near homes (time and place of biting e.g outdoors vs indoors), and to evaluate the potential for spread of other primate malarias in domestic settings. These findings will be useful for the control programme in designing vector control measures.

## Methods

### Selection of *P*. *knowlesi* cases for the study

The study was conducted in Kudat district which is located in the northeasternn tip of Sabah (6°53'14.35" N 116°49'25.10" E) and covers an area of approximately 1,300km^2^ with a population of 84,000 people of predominantly Rungus ethnicity (2010 National Census). The climate is tropical and the area is mainly coastal, with a maximum elevation of 250 metres above sea level. Forest cover is highly fragmented and substantial deforestation has occurred through conversion of forest to agricultural land [21, 22]. The majority of the population lives in small villages (mean population 160±15, S1 Table) and the main livelihood activities are small scale farming and plantation work.

*Plasmodium knowlesi* is the main cause of human malaria in Kudat and, due to this relatively high incidence, this area is the focus for a number of interdisciplinary studies on the biomedical, environmental and social risk factors for *P*. *knowlesi* (http://malaria.lshtm.ac.uk/MONKEYBAR). This includes a case control study, in which clinical malaria cases were recruited from the district hospital and visited at their homes within two weeks of initial diagnosis [22]. As there is mandatory reporting and referral of all malaria cases to the district hospital, the majority of symptomatic cases are captured by hospital systems.

**Fig 1:**
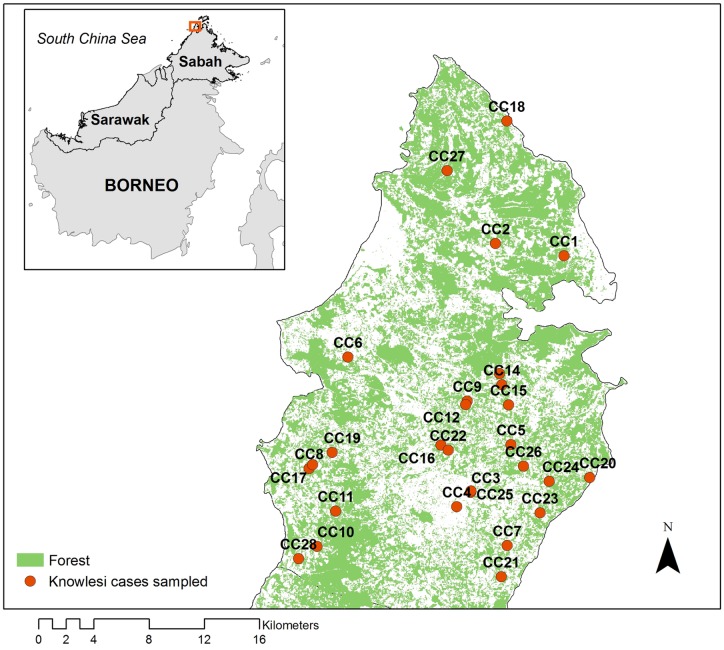
Distribution of *P*. *knowlesi* cases in Kudat District used in this study.

Approximately 180 *P*. *knowlesi* cases were identified through this active case surveillance between 2013–14; of which we randomly selected a subgroup of 28 for further entomological follow up (representing cases reporting February, July, 2014 from 23 different villages: Fig 1, S1 Table). The cases came from the age group 19–74 years old with a mean of 44. There was a preponderance of males amongst the cases, and many (78.6%) worked in agricultural sector, taking at least about 10–30 minutes to walk to their work place (Table 1)

**Table 1:** Demographic information of the cases and controls. The controls represent one individual selected randomly from each “control” household and is not age-based.

		Case		Control	
Demographic features		N	%	N	%
sex	Males	26	93	22	79
	Females	2	7	6	21
Mean age (range)		44 (19–74)		40 (2–72)	
Agricultural sector	Rubber estate	13	79	6	57
	Oil palm plantation	5		2	
	Coconut plantation	2		2	
	Vegetable garden	1		6	
	Fruit orchard	1		—	
Non-agricultural sector	House wife	1	14	4	32
	Others	3		5	
Others	Unspecified	1	7	—	11
	Not working	1		3	
Time to get to work place	0 (house wives, unemployed)	1	4	8	29
	10 min (agricultural)	15	54	5	18
	30 min (agricultural)	8	29	12	43
	2 hr (agricultural, construction worker)	1	4	2	7
	Unspecified	3	11	1	4

Additionally, a matched “control” household was recruited in the vicinity of the case household for study which shared similar environmental characteristics in terms of surrounding vegetation and terrain, but where no occupants had reported with any malaria infection within the study period as indicated by records from the hospital and interviewing the residents. From the group of potential “control” households identified in the vicinity, one was randomly selected using a random table. The final choice was also dependent on the owner’s agreement. Within two weeks of the case detection, the selection of control house was accomplished and entomological work initiated.

Data on the types of crops or vegetation surrounding households was collected, as well as the distance between each pair of case and control household (S2 Table). The mean distance between the case and the control houses was 255±48 m (18–1000 m). As the villages were generally small in area, occasionally a control house could be located in a neighbouring village. Of the 28 case-control household pairs, five pairs occurred within the same village.

### Collection of *Anopheles* using human landing catch

For all pairs of households, mosquito sampling was conducted by four workers each at case and control simultaneously on the same nights. At these pairs, sampling was conducted for one to three nights depending on the owner’s permission and logistic constraints.

Indoor collections were conducted at one station in the living room of houses (H), whereas outdoor collections were conducted at three selected stations (S1, S2 & S3) within the garden area surrounding the house. The distance of the stations from the house was 24±1.7 m for the case and 19±0.8 m for the control household. These outdoor stations were selected based on information provided by the family members about where they were most likely to spend time outdoors in the evening. Mosquitoes were baited using human landing catch (HLC) method, but only *Anopheles* spp were collected for further analysis, whilst other species were killed and discarded at the site. Here a volunteer collected mosquitoes by exposing his lower legs (from knee downwards) and collecting all mosquitoes that land upon them in a plastic specimen tube (2 cm diameter X 6 cm) which had a small piece of moist tissue at the bottom. Each station was manned by one person who would collect *Anopheles* for 12 hours straight (1800 to 0600 hr), and there was rotation of workers. The HLC workers at each case and control house were regularly monitored by a supervisor. The *Anopheles* were kept individually in a tube with a label which had information on the place, date, hour, location (indoors vs outdoors) and station of collection. The mosquito samples collected were recorded by hour in order to estimate the biting profile over course of night. The next morning the samples were taken to the laboratory to be processed.

### Identification of *Anopheles* species

*Anopheles* specimens were identified the next morning in the laboratory based on morphology characters using published identification keys. The key of Sallum et al. [23] was used for Leucosphyrus group, whereas keys developed by Rattanarithikul et al. [24] were used for other groups. The identified specimens were kept individually in a sterile 1.5mL microfuge tube and stored in -20°C until used for molecular analysis.

### Total DNA extraction and PCR of malaria parasites

Each *Anopheles* specimen was cut into two parts: head-thorax and abdomen, and placed separately inside an autoclaved mortar and the tissue homogenized using pestle. The total DNA was extracted from each part following the method of Phillips and Simon [25] and stored in -30°C until PCR analysis. Detection of malaria parasites in the *Anopheles* specimens was performed using the nested PCR *Plasmodium* genus-specific method described by Singh et al. [26]. When a sample was found positive for malaria parasites, a second nested PCR was performed to determine the *Plasmodium spp*. using species specific primers in singleplex PCR [4, 8, 26,27]. Primers of nine species of *Plasmodium* namely *P*. *coatneyi*, *P*. *inui*, *P*. *fieldi*, *P*. *cynomolgi*, *P*. *knowlesi*, *P*. *falciparum*, *P*. *vivax*, *P*. *malarie* and *P*. *ovale* were used in this study. All these species have been recorded in Malaysia although *P*. *ovale* is an imported species, while *P*. *knowlesi* is the prevalent simian malaria infecting man.

Both PCR 1 and PCR 2 were performed with 25μl final volume. The reaction components were prepared by mixing 5.0μl of 5X PCR buffer (Promega), 0.5μl of dNTPs (10mM) mixture (Promega), 3.0μl of 25mM MgCl_2_ (Promega), 1.0μl each of 10μM forward and reverse primers, 0.3μl of Taq DNA polymerase (5U/μl), 2.0μl of DNA template and sterile dH_2_O up to 25μl final volume. After the first PCR was completed, 2.0μl of the first PCR product was used as a template in the second PCR. The PCR conditions used were: an initial denaturation at 95°C for 5 min, followed by 35 cycles of 94°C for 1 min, annealing for 1 min and 72°C for 1 min, and a final extension at 72°C for 5 min. The annealing temperature was set based on the optimum temperature of the primers (S3 Table).

### Statistical analysis

Statistical analysis was conducted using R programming language for statistical analysis (version 3.2.2). Generalised linear mixed models (GLMM) were constructed to test for variation in the abundance of *Anopheles* between case and control houses, and indoor and peri-domestic settings. In the analysis, household type (case or control) and location (indoor or outdoor) were considered as fixed effects, while month, night and sampling station (site) as random effects. To identify the best model, both negative binomial and Poisson distributions, interaction between type and location, as well as zero inflation were fitted. Tukey's Post Hoc test was used to compare mean between fix effects (household type and location) as well interaction between these two effects.

We also analysed the proportion of mosquitoes that were caught feeding outdoors (P_o_), and the proportion of human exposure to *An*. *balabacensis* (P_e_).

The proportion of mosquitoes that were caught outdoors (P_o_) was calculated as

$$P_{o}=\frac{O_{18-06\;h}}{\left(O_{18-06\;h}+I_{18-06\;h}\right)}$$ (1)

where O and I are respectively the number of mosquitoes caught biting outdoors and indoors during 6 pm– 6 am.

From interviewing the residents and observation, more than 50% of the villagers would be indoors by 8 pm, and out the next morning by 5 am as they go to the plantations to work. The proportion of human exposure to *An*. *balabacensis* (P_e_) is thus calculated as the proportion of bites that happen outdoors during the time when people are likely to be outdoors, out of the sum of bites expected throughout the night as humans move between indoor and outdoor areas of their home:

$$P_{e}=\frac{O_{18-20,\;05\;h}}{\left(O_{18-20,\;05\;h}+I_{20-05\;h}\right)}$$ (2)

where O_18-20, 05 h_ represents the mosquitoes caught biting from 6–8 pm, and 5-6am, and I_20-5h_ represents the number caught indoors between 8 pm– 5 am. This would give a comparison between the proportion of bites people exposed to when outdoors between case and control households.

GLMM with a binomial distribution and a logit link function was used to obtain the binary estimates of P_o_ and P_e_. In these models, household type (case or control) was fitted as a fixed effect, while sampling station of the case as a random effect.

### Ethical clearance

This project was approved by the NMRR Ministry of Health Malaysia (NMRR-12-786-13048). All volunteers who carried out mosquito collections signed informed consent forms and were provided with antimalarial prophylaxis during participation. House owners also gave permission to use their houses for collection of mosquitoes.

## Results

### Employment in agricultural sector among case and control households

Among those who worked in the agricultural sector (Table 1), more than double the number cases were employed on rubber or oil palm plantations than controls, while more controls worked in the vegetable farms than cases.

### Composition of *Anopheles spp*.

A total of 793 *Anopheles* belonging to 12 species were caught during the period of study, with *An*. *balabacensis* being the dominant species (81% of total), followed by *An*. *maculatus*, *An*. *barbumbrosus*, and *An*. *donaldi* (Table 2). Overall, more *An*. *balabacensis* were caught at case (total 392 or 1.81 bites per man per night) than control houses (total 249 or 1.15 bites per man per night). Ten and 9 different *Anopheles* species were collected at case and control houses respectively, compared to only 6 and 2 indoor collections from case and control. Higher numbers were recorded at Kg. Tinukadan Laut (CC24), Kpg. Paradason B (CC26), Kpg. Nangka (CC5) (S1 Fig).

**Table 2:** *Anopheles* species caught outdoors and indoors at the case and control houses.

*Anopheles spp*.	Indoors		outdoors		total	%
	Case	Control	Case	Control		
*An*. *balabacensis* Baisas	18	16	374	233	641	80.83
*An*. *maculatus* Theobald	1	1	12	35	49	6.18
*An*. *barbumbrosus* Strickland & Chowdhury	2	0	24	11	37	4.67
*An*. *donaldi* Reid	1	0	7	26	34	4.29
*An*. *subpictus* Grassi	2	0	2	8	12	1.51
*An*. *tessellatus* Theobald	0	0	2	6	8	1.01
*An*. *peditaeniatus* Leicester	0	0	2	1	3	0.38
*An*. *whartoni* Reid	0	0	0	3	3	0.38
*An*. *umbrosus* Theobald	0	0	2	0	2	0.25
*An*. *indefinitus* Ludlow	1	0	1	0	2	0.25
*An*. *kochi* Dönitz	0	0	1	0	1	0.13
*An*. *baezai* Gater	0	0	0	1	1	0.13
Total species	6	2	10	9	-	-
Total individuals	25	17	427	324	793	100.00
Percentage	5.5	5.0	95.5	95.0		

**Fig 2:**
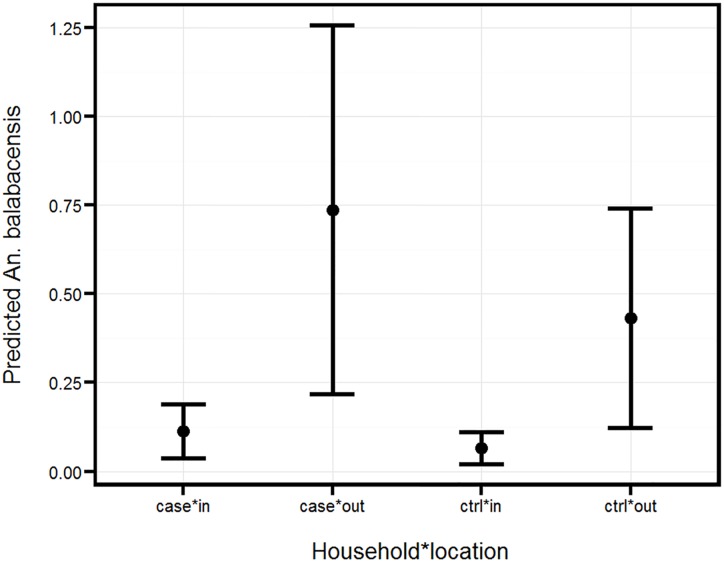
Mean number ± SE of *An*. *balabacensis* as predicted by GLMM. The value represents number caught per house per night from the case and control houses outdoors and indoors.

GLMM analysis indicated that the negative binomial distribution gave a better fit than Poisson distribution. The log-likelihood values were for negative binomial and Poisson distribution -513 vs -529 respectively, while the Akaike information criterion (AIC) values were 1043 vs 1068. Adopting the model with a negative binomial distribution, the abundance of *An*. *balabacensis* was found to vary significantly between case and control (case 0.28±0.194 vs control 0.17±0.127, z = 4.62, p<0.001), and between the surrounding peri-domestic area and inside the house (0.56± 0.394 versus 0.09±0.063, z = 9.09, p<0.001) (Fig 2). The interaction between house type and location was not significant (z = 0.8, P>0.05)

### Biting time of *An*. *balabacensis*

**Fig 3:**
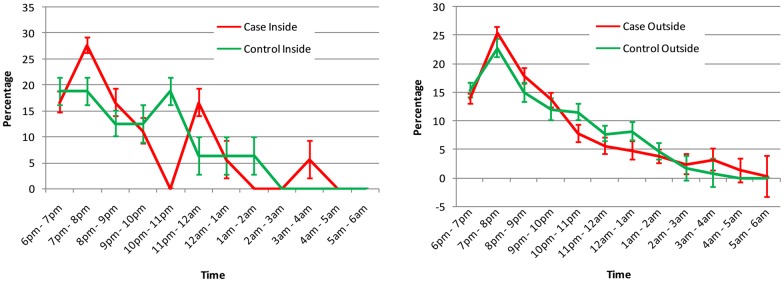
Biting time of *An*. *balabacensis* mosquitoes caught indoors and outdoors at the case and control houses. Data represent percentage of *An*. *balabacensis* (out of total collected) for each hour for the whole study period. Error bar represent 95% standard error of the mean.

More than 50% of *An*. *balabacensis* mosquitoes were caught biting in the early evening (6pm - 9pm) with the peak hour between 7pm - 8pm (Fig 3). After 8pm, the number rapidly decreased with approximately 84% of the total nightly catch being accumulated by midnight. Nevertheless, one or two individuals of *An*. *balabacensis* could still be caught until dawn.

In general, the proportion of bites taken by *An*. *balabacensis* outdoors (P_o_) was very high (>95%), and did not vary between case and control households (Table 3). Similarly the proportion of human exposure to bites (P_e_) did not vary between case and control households.

**Table 3:** GLMM analysis of proportion of *An*. *balabacensis* biting occurring outdoors (P_o_) and proportion of human outdoor exposure to mosquito bites (6–8 pm and 5–6 am) (P_e_). In the analyses household (case or control) was considered as the fixed effect.

Parameter	Coefficients of GLMM		Proportion of biting for household type		Tukey’s test case vs control
	Intercept	Household	Case	Control	
P_o_	3.343	-0.258	0.9659	0.9563	p>0.05
P_e_	4.074	-0.469	0.9833	0.9735	p>0.05

### Infection rate of *An*. *balabacensis*

A total of 793 *Anopheles* individuals were tested by molecular method and only ten *An*. *balabacensis* (out of 641 or 1.56%) were found to be positive for malaria parasites. Seven of them were caught at case houses (6 outdoors and one indoors), and 3 at control houses (all outdoors, Table 4). All these mosquitoes were found to be positive for simian malaria parasites (*P*. *coatneyi*, *P*. *inui*, *P*.*cynomologi & P*. *fieldi*); but none were with either the dominant zoonotic parasite reported in the area (*P*. *knowlesi*) or any human-specific *Plasmodium*. Ninety percent of infected *An*. *balabacensis* was caught biting outdoors between 6pm - 10pm (9 out of 10), and with one infected individual caught between 1am - 2am.

**Table 4:** *Plasmodium spp*. found in the infected *An*. *balabacensis* by PCR analysis.

*Plasmodium spp*.	No. infected	House	Site	Time	Reference No.
*P*. *coatneyi*	1	Control	Outdoor	7pm	CC16
*P*. *inui*	2	Case	Outdoor	7pm	CC23
		Case	Outdoor	1am	CC24
*P*. *cynomolgi*	3	Case	Outdoor	8pm	CC8
		Case	Outdoor	9pm	CC26
		Control	Outdoor	7pm	CC24
*P*. *inui* + *P*. *fieldi*	2	Case	Outdoor	6pm	CC15
		Case	Indoor	7pm	CC15
*P*. *inui* + *P*. *cynomolgi*	1	Case	Outdoor	9pm	CC23
*P*. *inui* + *P*. *fieldi* + *P*. *cynomolgi*	1	Control	Outdoor	8pm	CC15

The proportion of infected *An*. *balabacensis* caught from case houses (7/392 or 1.79%) was slightly higher than at control houses (3/249 or 1.20%). However, the sample sizes were too low for any robust statistical analysis of differences.

## Discussion

We conducted a randomized case-control field study to test the hypothesis that there is an association between *P*. *knowlesi* infection risk and higher exposure to mosquito vectors in peri-domestic (outdoors surrounding houses) and within domestic (inside house) settings. Although 12 *Anopheles* species were caught by HLC, only 4 were detected in reasonably high abundance: *An*. *balabacensis*, *An*. *maculatus*, *An*. *barbumbrosus* and *An*. *donaldi*. Of these, only *An*. *balabacensis* and *An*. *donaldi* have been previously implicated as malaria vectors of human malaria in Sabah [18,28]. In contrast, *An*. *maculatus* is the main malaria vector of human malaria in peninsular Malaysia [29]. *Anopheles balabacensis* appears to be a widespread species found in almost all sites, although significantly high numbers were caught in Kpg Tinukadan Laut (CC24) especially in those areas near to forest fringes. About 95% of *An*. *balabacensis* was caught outdoors, similar to what was previously recorded (a ratio of outdoor:indoor catch of 24:1) in Kuala Penyu, another district in Sabah [28]. A recent study conducted in Banggi Island situated north of Sabah and in Kg Paradason in the interior of Kudat district [10] also revealed that *An*. *balabacensis* was the predominant species collected, followed by *An*. *donaldi* in both sites. However, the next most abundant species was *An*. *vagus*, in Banggi, but *An*. *barbirostris* group in Kg Paradason. *Anopheles maculatus* was not caught in Banggi.

GLMM analysis indicated a significant difference between the number of vectors caught at case and control houses, and between outdoor and indoor catches. The primary vector of *P*. *knowlesi* in the area, *An*. *balabacensis*, was present at higher abundance at households where cases were reported, which would suggest a higher risk at the case houses. Furthermore, as 90% of the infected mosquitoes were caught outdoors, it is likely that peri-domestic infection is an important risk factor. Although the indoor number of infective mosquitoes caught was small, getting infected indoors cannot be discounted.

In Sarawak, it was postulated that humans were likely to acquire infection of *P*. *knowlesi* from being bitten by infected *An*. *latens* while hunting in the forest or as they return from the farm around dusk since in their study no infective mosquitoes were obtained from the village [13]. However, in Sabah clustering of cases among family members have been reported and they postulated that people could be infected around their homes [30]. A recent study also in Sabah showed the presence of asymptomatic cases of *P*. *knowlesi* occurring among the community in Kudat [31]. Thus, the result of this study seems to support the hypothesis that it is also possible for people to be infected in and around their homes. Although *An*. *balabacensis* is highly exophagic with only one infected individual found indoors we should not dismiss the fact that the possibility of indoor infection does exist. Thus we need to possibly expand our paradigm about transmission of *P*. *knowlesi* to include the possibility of peri-domestic infection, and conduct further studies to evaluate simultaneously the infection risk in and around households, as well as in forest areas, so the relative contribution of all these routes could be formally quantified.

Many areas in Kudat district have undergone deforestation and clearance of vegetation for crop plantations, but it appears that *An*. *balabacensis* has remained the dominant species, with the exception of Kinabatangan area of Sabah where *An*. *balabacensis* was replaced by *An*. *donaldi* as main malaria vector as a result of deforestation and malaria control activity [18]. This suggests that the abundance of *An*. *balabacensis* in Kudat district was not greatly affected by the environmental changes. The impact of forest disturbance such as logging has been shown to increase the abundance of this disease vector and may partly explain the rapid rise in *P*. *knowlesi* cases in Sabah [32].

The feeding time of *An*. *balabacensis* appears to have changed since late 1960s when most of the area in Kudat district was still covered with forest. A study conducted then [29] showed that *An*. *balabacensis* was actively biting human at late night (10pm onwards), compared to early night with peak hour between 7pm to 8pm recorded now. In fact, this species starts biting human outdoors as soon as it starts to get dark. This change in feeding time could be due to *An*. *balabacensis* adapting to more people staying closer to forest fringe as more forested areas are cleared for crop plantation and housing. This could also be due to the introduction of insecticide treated bednets. Further study will be needed to confirm this.

Although we did not obtain an *An balabacensis* individual infected with *P*. *knowlesi*, as only ten individuals were found *Plasmodium* positive albeit for other simian malarias, this could be a sampling error. As such, we are unable to make a conclusive prediction about infection risk at case households. Given the generally low rates of *P*. *knowlesi* infection in the vector (eg 13/1482 or 0.88%, data also collected in Kudat) [10], thousands of samples are needed to obtain strong evidence to show that *P*. *knowlesi* was not present, and/or to compare infection rates between case and control households. Furthermore, as the densities of vectors in these settings are generally low, it would not have been feasible to achieve this sample size within the one year time span that the case control study was running. What data collected here do show however is that the primary vector is present at higher abundance in peridomestic settings where cases are reporting, on which basis the possibility of peridomestic transmission cannot be dismissed. This also indicates that people are routinely exposed to a variety of different primate malarias around their home; but that to date, only a couple (*knowlesi* and *cynomologi*) seem capable to causing any clinical infection. More research needs to be carried out to determine why these two primate malarias succeeded where the others fail so we can be proactive in the fight against future new simian malarias infecting man.

The difference between bites per man per night between case and control houses is 0.66 (1.81–1.15) which works out to be 241more bites per person in a year for the case house. Since the infective proportion of vector is 0.88 [10], this is equivalent to a higher entomological inoculation rate (EIR) of 2.12. In addition, more than double the number cases worked in rubber or oil palm plantations than controls. Perhaps these two factors may help explain why there was *P*. *knowlesi* infection in the case houses. However more research is needed to validate this.

As most *P*. *knowlesi* cases have been recorded from villages close to where macaques abound, and given that the primary vectors species, *An balabacensis* bite outdoors, a new paradigm in managing this malaria is needed. More attention should be focused on the ecology and biology of *An*. *balabacensis* in order to develop more effective control methods if the control or elimination of *P*. *knowlesi* malaria in Kudat district is to be successful. The current malaria control programme using ITNs might not have the desired impact as this species is mainly an exophagic species, and infection is more likely to occur outdoors in peri-domestic settings, in plantations and forest.

## Supporting Information

S1 Table General information of the case and control houses. (PDF)

S2 Table General condition and habitat at the case and control houses. (PDF)

S3 Table Sequences and annealing temperature of each pair of the primers. (PDF)

S1 Fig *An*. *balabacensis* (bites/man/night) recorded in each of case (red) and control (green) houses. (TIF)

S1 Checklist STROBE Checklist. (PDF)
