# Supplementary material for: Investigating the Contribution of Peri-domestic Transmission to Risk of Zoonotic Malaria Infection in Humans
Source: PLoS Negl Trop Dis. 2016 Oct 14;10(10):e0005064. doi: 10.1371/journal.pntd.0005064 (PMC5065189; doi:10.1371/journal.pntd.0005064)
Supplement: S1 Table — (PDF) [file pntd.0005064.s001.pdf]

S1 Table. General information of the case and control houses.

Ref.	Village	Coordinates	Pop of village	Distance (m) between case & control houses	Date *P. knowlesi* case reported	Type	Sampling date	Number of sampling nights	Number of collectors
CC1	Kpg. Lau San	N: 06.90645°<br>E: 116.82725°	386	83	4/2/2014	Case<br>Control	17/02/14	1	4
CC2	Kpg. Tai Cheong	N: 06.91458°<br>E: 116.78236°	93	145	9/2/2014	Case<br>Control	21/02/14	1	4
CC3	Kpg. Pomonsukan	N: 06.75201°<br>E: 116.76631°	179	42	21/02/14	Case<br>Control	5/3/2014	1	4
CC4	Kpg. Radtak	N: 06.74268°<br>E: 116.75720°	176	18	10/2/2014	Case<br>Control	1/3/2014	1	4
CC5	Kpg. Nangka	N: 06.78325°<br>E: 116.79260°	145	171	17/03/14	Case<br>Control	31/03/14 - 02/04/14	3	4
CC6	Kpg. Lajung Kobibi	N: 06.84050°<br>E: 116.68612°	143	983	26/03/14	Case<br>Control	07/04/14 - 09/04/14	3	4
CC7	Kpg. Kindangan	N: 06.71749°<br>E: 116.79010°	137	93	4/4/2014	Case<br>Control	14/04/14 & 15/04/14	2	4
CC8	Kpg. Jagil	N: 06.76780°<br>E: 116.66087°	113	379	5/4/2014	Case<br>Control	16/04/14 & 17/04/14	2	4
CC9	Kpg. Pinturu	N: 06.81172°<br>E: 116.76406°	104	41	11/4/2014	Case<br>Control	23/04/14 & 24/04/14	2	4
CC10	Kpg. Rita	N: 06.71695°<br>E: 116.66600°	86	36	13/04/14	Case<br>Control	28/04/14 & 29/04/14	2	4
CC11	Kpg. Minikodong	N: 06.73986°<br>E: 116.67802°	198	100	15/04/14	Case<br>Control	30/04/14 & 01/05/14	2	4
CC12	Kpg. Pinturu	N: 06.80947°<br>E: 116.76294°	104	121	23/04/14	Case<br>Control	05/05/14 & 06/05/14	2	4
CC13	Kpg. Lodung	N: 06.82951°<br>E: 116.78516°	109	67	25/04/14	Case<br>Control	07/05/14 & 08/05/14	2	4
CC14	Kpg. Lodung	N: 06.82239°<br>E: 116.78669°	109	163	25/04/14	Case<br>Control	12/05/14 & 13/05/14	2	4

Ref.	Village	Coordinates	Pop of village	Distance (m) between case & control houses	Date *P. knowlesi* case reported	Type	Sampling date	Number of sampling nights	Number of collectors
CC15	Kpg. Tomohon	N: 06.80918°<br>E: 116.79096°	63	128	28/04/14	Case<br>Control	14/05/14 & 15/05/14	2	4
CC16	Kpg. Narandang	N: 06.78297°<br>E: 116.74675°	235	104	12/5/2014	Case<br>Control	19/05/14 & 20/05/14	2	4
CC17	Kpg. Jagil	N: 06.77024°<br>E: 116.66312°	113	376	5/5/2014	Case<br>Control	21/05/14 & 22/05/14	2	4
CC18	Kpg. Suang Pai	N: 06.99454°<br>E: 116.78979°	324	1050	19/05/14	Case<br>Control	26/05/14 & 27/05/14	2	4
CC19	Kpg. Rondoman	N: 06.77824°<br>E: 116.67590°	160	286	22/05/14	Case<br>Control	28/05/14 & 29/05/14	2	4
CC20	Kpg. Togumamal Laut	N: 06.76173°<br>E: 116.84392°	51	73	26/05/14	Case<br>Control	04/06/14 & 05/06/14	2	4
CC21	Kpg. Kondopi	N: 06.69707°<br>E: 116.7862°	123	62	26/05/14	Case<br>Control	09/06/14 & 10/06/14	2	4
CC22	Kpg. Narandang	N: 06.77977°<br>E: 116.75154°	235	249	1/6/2014	Case<br>Control	11/06/14 & 12/06/14	2	4
CC23	Kpg. Mambatu Laut	N: 06.73880°<br>E: 116.81166°	135	74	2/6/2014	Case<br>Control	16/06/14 & 17/06/14	2	4
CC24	Kpg. Tinukadan Laut	N: 06.75930°<br>E: 116.81754°	43	76	10/6/2014	Case<br>Control	18/06/14 & 19/06/14	2	4
CC25	Kpg. Pomonsukan	N: 06.75307°<br>E: 116.76637°	179	84	11/6/2014	Case<br>Control	23/06/14 & 24/06/14	2	4
CC26	Kpg. Paradason B	N: 06.76915°<br>E: 116.80067°	65	30	17/06/14	Case<br>Control	25/06/14 & 26/06/14	2	4
CC27	Kpg. Longgom Besar	N: 06.96205°<br>E: 116.75076°	244	38	11/7/2014	Case<br>Control	21/07/14 & 22/07/14	2	4
CC28	Kpg. Kodiung Darat	N: 06.70893°<br>E: 116.65401°	173	40	21/07/14	Case<br>Control	23/07/14 & 24/07/14	2	4
	Mean ± SE		160 ± 15	255 ± 48					
