# Supplementary material for: Investigating the Contribution of Peri-domestic Transmission to Risk of Zoonotic Malaria Infection in Humans
Source: PLoS Negl Trop Dis. 2016 Oct 14;10(10):e0005064. doi: 10.1371/journal.pntd.0005064 (PMC5065189; doi:10.1371/journal.pntd.0005064)
Supplement: S2 Table — (PDF) [file pntd.0005064.s002.pdf]

S2 Table. General condition and habitat at the case and control houses.

Description of surroundings	Number of houses fitting the description	
	case	control
**General condition at outside of the house (within 50m radius)**		
• clean (without rubbish or bushes surrounding the house)	16	13
• dirty (with rubbish, plastic, metal or zinc outside the house but not surrounded with bushes)	2	4
• dirty (with rubbish, plastic, metal or zinc and surrounded with bushes)	10	11
**Type of habitat outside of the house (within 50m radius)**		
• flowers on the porch of the house	14	12
• crop garden (banana, papaya, pineapple, tapioca, corn or vegetables)	11	11
• the house area surrounded with trees	19	14

• Mixture of either oil palm, rubber or coconut trees	18	19
**Presence of water body (within 100m radius)**		
• River	16	14
• Lake	1	1
• pond	2	6
• water holes	1	1
• paddy field	1	1
• drain	1	1
• none	8	6

Note: All case and control houses were surrounded by trees of various species and sizes, including rubber and coconut trees within a 50m radius from their houses. The residents of some of these houses also planted flowering plants and cash crops (eg. banana, papaya, pineapple, tapioca, corn and vegetables) outside their houses for their own consumption. In some villages (Kg. Tomohon), at least one type of permanent water body including rivers, lakes and ponds, were present within a 100 m radius of focal houses. Many of the households also stored water in big plastic containers with lids (1,800 litres) supplied by the government, although there were owners who had left the containers uncovered.
