# Supplementary material for: Investigating the Contribution of Peri-domestic Transmission to Risk of Zoonotic Malaria Infection in Humans
Source: PLoS Negl Trop Dis. 2016 Oct 14;10(10):e0005064. doi: 10.1371/journal.pntd.0005064 (PMC5065189; doi:10.1371/journal.pntd.0005064)
Supplement: S3 Table — (PDF) [file pntd.0005064.s003.pdf]

S3 Table. Sequences and annealing temperature of each pair of the primers.

Target	*Plasmodium*	Primer sets used in nested PCR	Primer set and name		Sequence (5' - 3')	Annealing temp.	Size of PCR product (bp)
SSU-rRNA	*Plasmodium* genus	Sets 1+ 2	set 1	rPLU1[*]	TCAAAGATTAAGCCATGCAAGTGA	55°C	1640
				rPLU5[*]	CCTGTTGTTGCCTTAAACTCC		
			set 2	rPLU3[*]	TTTTTATAAGGATAACTACGGAAAAGCTGT	62°C	240
				rPLU4[*]	TACCCGTCATAGCCATGTTAGGCCAATACC		
	P. coatneyi	Sets 1+ 3	set 3	PctF1[+]	CGCTTTTAGCTTAAATCCACATAACAGAC	62°C	504
				PctR1[+]	GAGTCCTAACCCCGAAGGGAAAGG		
	P. inui	Sets 1+ 4	set 4	PinF2[+]	CGTATCGACTTTGTGGCATTTTTCTAC	60°C	479
				INAR3[+]	GCAATCTAAGAGTTTTAACTCCTC		
	P. fieldi	Sets 1+ 5	set 5	PfldF1[+]	GGTCTTTTTTTTGCTTCGGTAATTA	66°C	421
				PfldR2[+]	AGGCACTGAAGGAAGCAATCTAAGAGTTTC		
	P. cynomolgi	Sets 1+ 6	set 6	CY2F[+]	GATTTGCTAAATTGCGGTCG	60°C	137
				CY4R[+]	CGGTATGATAAGCCAGGGAAGT		
	P. knowlesi	Sets 1+ 7	set 7	PkF1140[#]	GATTCATCTATTAAAAATTTGCTTC	50°C	424
				PkR1550[#]	GAGTTCTAATCTCCGGAGAGAAAAGA		
	P. falciparum	Sets 1+ 8	set 8	NewPLFshort[$]	CTATCAGCTTTTGATGTTAG	53°C	370
				FARshort[$]	GTTCCCCTAGAATAGTTACA		
	P. vivax	Sets 1+ 9	Set 9	NewPLFshort[$]	CTATCAGCTTTTGATGTTAG	53°C	476
				VIRshort[$]	AAGGACTTCCAAGCC		
	P. malariae	Sets 1+ 10	Set 10	NewPLFshort[$]	CTATCAGCTTTTGATGTTAG	53°C	241
				MARshort[$]	TCCAATTGCCTTCTG		
	P. ovale	Sets 1+ 11	Set 11	NewPLFshort[$]	CTATCAGCTTTTGATGTTAG	53°C	407
				OVRshort[$]	AGGAATGCAAAGARCAG		

[*]Singh et al., 1999; [#]Imwong et al., 2009; [+]Lee at al., 2011; [$]Ta et al., 2014
