# Supplementary figures and images for: Investigating the Contribution of Peri-domestic Transmission to Risk of Zoonotic Malaria Infection in Humans
Source: PLoS Negl Trop Dis. 2016 Oct 14;10(10):e0005064. doi: 10.1371/journal.pntd.0005064 (PMC5065189; doi:10.1371/journal.pntd.0005064)

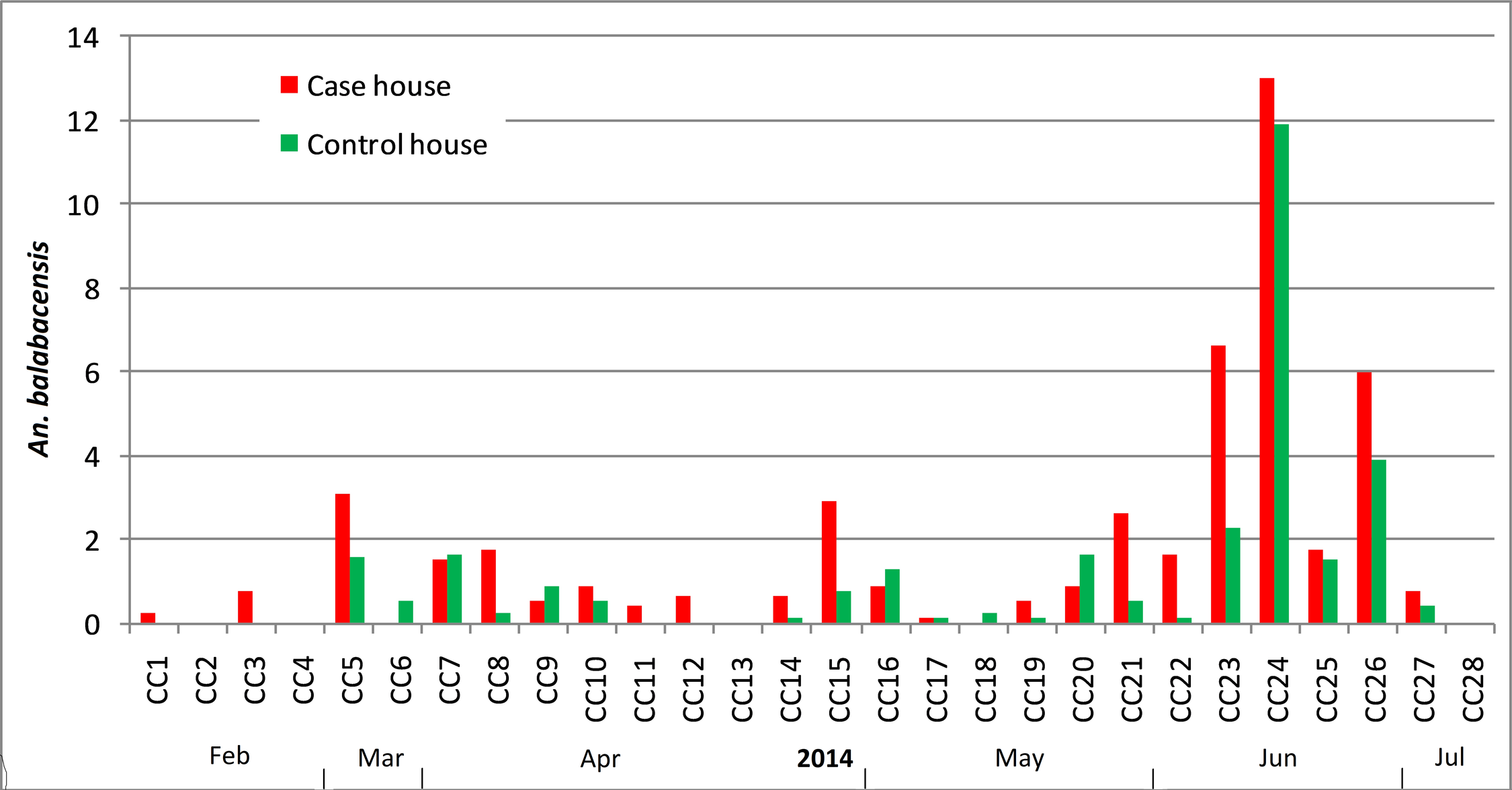

Supplement: S1 Fig — (TIF) [file pntd.0005064.s004.tif]
